# Alteration of vasopressin-aquaporin system in hindlimb unloading mice

**DOI:** 10.3389/fphys.2025.1535053

**Published:** 2025-04-15

**Authors:** Marianna Ranieri, Maria Venneri, Giuseppina Storlino, Angela Ferrulli, Mariagrazia D’Agostino, Mariangela Centrone, Annarita Di Mise, Roberta Zerlotin, Grazia Tamma, Maria Grano, Giovanna Valenti

**Affiliations:** ^1^ Department of Biosciences, Biotechnologies and Environment, University of Bari, Bari, Italy; ^2^ Istituti Clinici Scientifici Maugeri SPA SB IRCCS, Bari, Italy; ^3^ Department of Clinical and Experimental Medicine, University of Foggia, Foggia, Italy; ^4^ Department of Biotechnology and Biosciences, University of Milano-Bicocca, Milan, Italy; ^5^ Department of Precision and Regenerative Medicine and Ionian Area, University of Bari, Bari, Italy

**Keywords:** AQP2, vasopressin (ADH), vasopressin type 2 receptor, hindlimb suspension (unloading), microgravity, microRNA, calcium-sensing receptor (CaSR)

## Abstract

Murine hindlimb unloading (HU) is considered a model of choice for simulating the physiological effects of microgravity on several functions, including fluid and electrolyte homeostasis. Microgravity causes changes in blood redistribution, modulating vasopressin secretion, a major hormone controlling water reabsorption through the vasopressin-sensitive water channel AQP2. In this study, mice were hindlimb suspended over 4 weeks or rested in the ground as controls, and vasopressin levels, along with renal aquaporins expression were investigated. Copeptin, a stable precursor of the hormone vasopressin, significantly increased as early as 1 week of unloading which correlated with a significant increase in AQP2 total protein expression and decrease in serum osmolality, suggesting early activation of the vasopressin/AQP2 axis in this model. Conversely, in 4 weeks HU suspended mice, copeptin decreased significantly and both AQP2 mRNA and AQP2 total protein expression were significantly reduced. Consistent with a downregulation of the vasopressin/AQP2 axis an increase in serum osmolality was observed at 4 weeks HU. The basolateral water channels AQP3 and AQP4 were, on the other hand, unaffected. Immunolocalization studies confirmed reduced expression of AQP2 in renal collecting ducts of HU mice at 4 weeks. A significantly increased amount of the expressed AQP2 was found phosphorylated at Ser261, a site regulating AQP2 protein stability and degradation. In line, p38-MAPK, committed to phosphorylate Ser261 and to increase miR137 expression, an AQP2 mRNA-targeted microRNA, was significantly increased in HU, suggesting that reduced AQP2 expression was mainly due to increased protein degradation and downregulation of AQP2-mRNA translation. Our results suggest that vasopressin/AQP2 axis is upregulated as early as 1 week and may be involved in the antidiuretic response also observed in early spaceflight period in astronauts. Contrariwise, the vasopressin-AQP2 system is downregulated after 4 weeks HU, likely to counteract the persistent central venous pressure due to cephalic shift of fluids.

## Introduction

Body fluid regulation is affected by microgravity causing reno-vascular changes (Olde Engberink et al., 2023). In particular, microgravity causes fluid redistribution from the legs to the upper part of the body, which impacts kidney function and volume homeostasis (Moore and Thornton, 1987; Thornton et al., 1992; Olde Engberink et al., 2023; Beysens and Loon, 2015). The major hormone regulating body fluid distribution is the antidiuretic hormone vasopressin. Vasopressin is secreted in response to an increase in plasma osmolality or decreased extracellular volume. In the kidney, it binds its receptor (vasopressin V2 receptor) in the collecting duct principal cells, initiating a signal transduction cascade leading to redistribution of aquaporin-2 (AQP2) water channels to the luminal plasma membrane, thereby increasing water permeability. Thus, water can be reabsorbed in the renal collecting duct to fine-tune water balance and plasma osmolality (Centrone et al., 2022; Ranieri et al., 2019; Kharin and Klussmann, 2024). In the light of the above considerations, is of interest to study the modulation of the vasopressin-aquaporin system occurring during exposure to microgravity.

Several models for simulating the effects of microgravity exist for various species. In humans, a well-established terrestrial model is head-down bed rest which induces a cephalic fluid shift and results in the loss of impact loading on the musculoskeletal system (Convertino et al., 1990; Barkaszi et al., 2022; Le Roux et al., 2022; Qaisar et al., 2020). Similarly, water immersion of humans causes a rapid cephalad fluid shift, unloading of the immersed systems (Watenpaugh, 2016; Valenti et al., 2006).

Hindlimb unloading (HU) of rodents in a head-down position, is an animal model to simulate the physiological effects of microgravity on ground (Globus and Morey-Holton, 2016; Morey-Holton and Globus, 2002). The HU model was developed in the 1980s to study mechanisms, responses, and treatments for the adverse consequences of space flight. In this model, the hindlimbs of rodents are elevated to produce a 30° head-down tilt, which results in a cephalad fluid shift (Morey-Holton and Globus, 2002). Although the rat hindlimb unloading (HU) model was initially developed in particular to study alterations of the musculoskeletal and cardiovascular systems caused by weightlessness (Morey, 1979), several studies have demonstrated that this model can be used to study alterations of many physiological properties including fluid and electrolyte homeostasis (Globus and Morey-Holton, 2016; Morey-Holton and Globus, 2002; Chung et al., 2012). However, data obtained from hindlimb unloading experiments must be interpreted with caution since in humans the entire body is unloaded during spaceflight, whereas the forelimbs, head, and upper back remain weightbearing in hindlimb-unloaded animals. Moreover, in HU fluid shifts may be greater in hindlimb-unloaded rodents because the animal is quadruped. Despite these cautions, studies in hindlimb-unloaded rats have been instrumental in defining the time course and mechanisms of physiological changes that occur due to unloading. A previous study using HU rat model examined time dependent alterations of vasopressin for 1 (HU1), 7 (HU7), 14 days (HU14) showing a peak value of both plasma vasopressin levels and AQP2 at HU7. Alterations were however restored at HU14 (Chung et al., 2012) suggesting that in this model, vasopressin system may require 2 weeks for adaptation to microgravity. These results differ however from another study showing higher vasopressin levels at HU14 (Chung et al., 2012). In the present contribution, mice were hindlimb suspended over 4 weeks or rested in the ground as controls, and vasopressin levels along with renal aquaporins expression was investigated. For the first time in rodent HU models, plasma copeptin levels were measured as validated surrogate for plasma vasopressin levels. Measurement of copeptin is increasingly preferred over measurement of vasopressin because of the technical constraints of the conventional assays for vasopressin (Heida et al., 2017).

Obtained results indicate that in mice, while vasopressin-AQP2 axis is upregulated as early as 1 week, 4 weeks hindlimb unloading results in downregulation of the vasopressin - AQP2 system, likely as a consequence of the persistent cephalic shift of fluids.

## Results

### Evaluation of serum copeptin, aquaporins expression and serum osmolality in HU mice

Serum copeptin levels were measured (ELISA kit) as validated surrogate for serum vasopressin levels.

**FIGURE 1:**
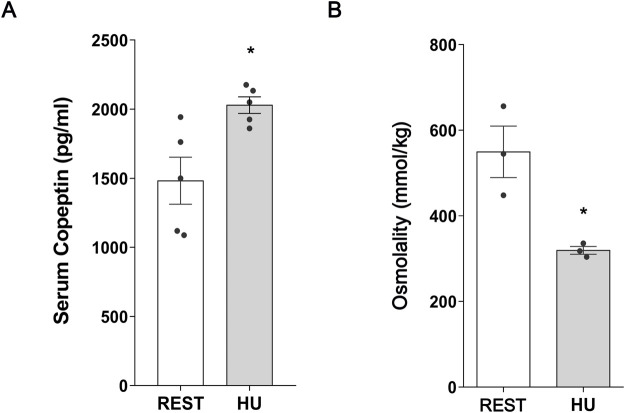
**(A)** Serum Copeptin in 1 week hindlimb unloading rodent model (HU) compared to control (REST). ELISA assay was performed on serum obtained from HU and REST mice. Data are expressed as mean ± S.E.M. (pg/mL). Statistical analysis was done using Unpaired t-test (*p < 0.05). **(B)** Serum Osmolality in hindlimb unloading rodent model (HU) compared to control (REST). Data are expressed as mean ± S.E.M. (mmol/kg). Statistical analysis was done using Unpaired t-test (*p < 0.05).

At 1 week HU suspension, copeptin levels were significantly increased in HU mice compared to the Rest mice (HU: 2,029 ± 59.98 pg/mL vs. REST: 1,483 ± 170.1 pg/mL; *p = 0.016*) (Figure 1A).

**FIGURE 2:**
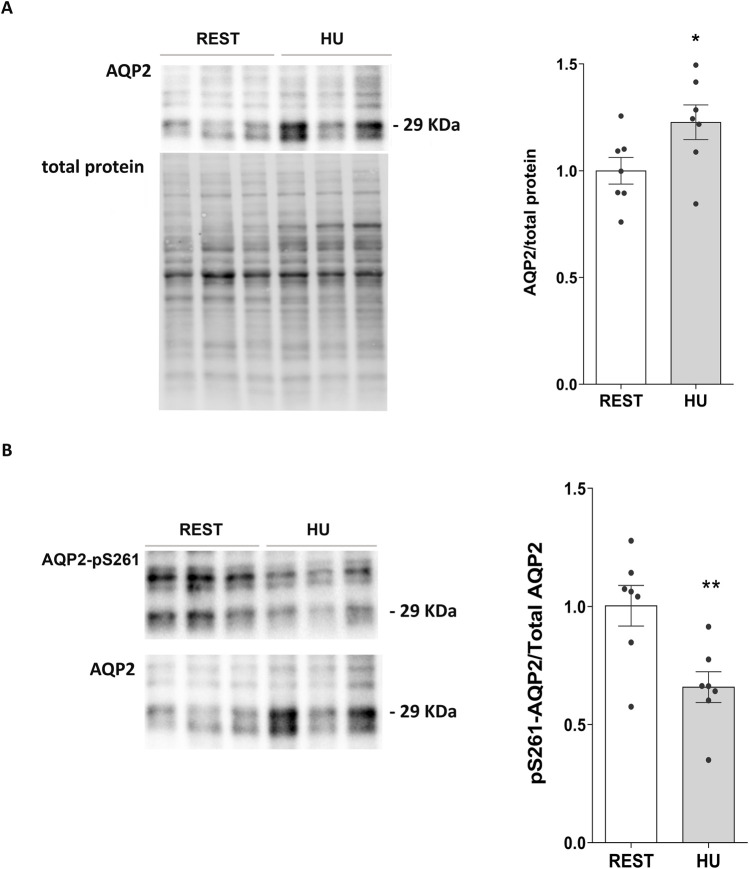
**(A)** Expression of AQP2 in 1 week HU and REST mice. Kidneys from HU and REST mice were lysed, and immunoblotting experiments were performed using specific antibodies against total AQP2. Densitometric analysis of total AQP2 bands normalized to the total protein content is reported in the histogram. Data are expressed as mean ± S.E.M. (O.D.). Statistical analysis was done using Unpaired t-test (*p < 0.05). **(B)** Expression of AQP2 in 1 week HU and REST mice. Kidneys from HU and REST mice were lysed, and immunoblotting experiments were performed using specific antibodies against total AQP2. Densitometric analysis of total AQP2 bands normalized to the total protein content is reported in the histogram. Data are expressed as mean ± S.E.M. (O.D.). Statistical analysis was done using Unpaired t-test (**p < 0.01).

In line with this data, total AQP2 expression in kidney homogenate from 1 week HU suspended mice significantly increased with respect to Rest mice (HU: 1.227 ± 0.081 O.D. vs. REST: 1.000 ± 0.062 O.D.; *p = 0.047*) (Figure 2A), along with a significant reduction in pS261-AQP2 (HU: 0.659 ± 0.065 O.D. vs. REST: 1.000 ± 0.086 O.D.; *p = 0.008*) (Figure 2B), a phosphorylation site crucial for the degradation processes via proteasome (Nedvetsky et al., 2010; Isobe et al., 2017). This indicates that AQP2 is less degraded at early timepoint of HU.

In agreement with the increase in copeptin levels, serum osmolality was significantly reduced in HU mice, reflecting higher water reabsorption resulting in reduction in serum osmolality (HU: 319.3 ± 9.26 mmol/kg vs. REST: 549.7 ± 60.09 mmol/kg; *p = 0.019*) (Figure 1B).

**FIGURE 3:**
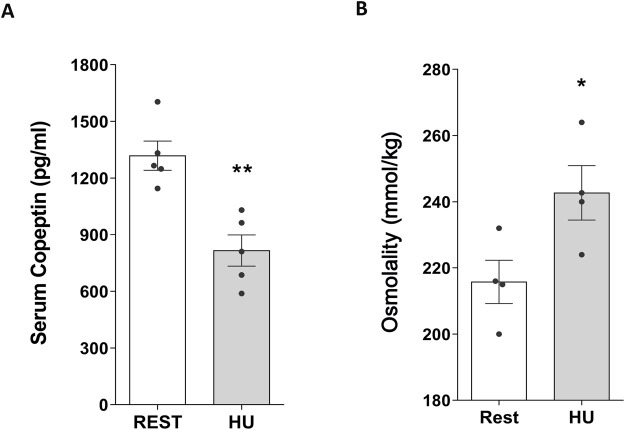
**(A)** Serum Copeptin in 4 weeks hindlimb unloading rodent model (HU) compared to control (REST). ELISA assay was performed on serum obtained from HU and REST mice. Data are expressed as mean ± S.E.M. (pg/mL). Statistical analysis was done using Unpaired t-test (**p < 0.01). **(B)** Serum Osmolality in hindlimb unloading rodent model (HU) compared to control (REST). Data are expressed as mean ± S.E.M. (mmol/kg). Statistical analysis was done using Unpaired t-test (*p < 0.05).

Conversely, at 4 weeks HU, copeptin levels were significantly decreased in HU mice compared to Rest mice (HU: 815.9 ± 82.49 pg/mL vs. REST: 1,319.0 ± 77.29 pg/mL; *p = 0.002*) (Figure 3A), along with a significant increase in serum osmolality (HU: 242.7 ± 8.22 mmol/kg vs. REST: 215.8 ± 6.54 mmol/kg; *p = 0.043*) suggesting that mice are in negative fluid balance at 4 weeks of suspension (Figure 3B).

**FIGURE 4:**
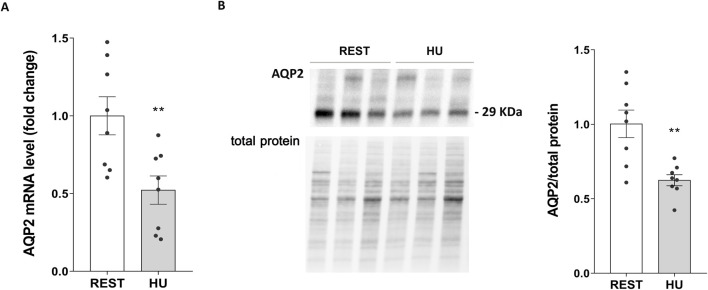
**(A)** AQP2 mRNA levels in 4 weeks HU and REST mice. RNA was extracted from HU and REST mice kidneys as described in Methods Section. Data are expressed as mean ± S.E.M. Statistical analysis was done using Unpaired t-test (**p < 0.01). **(B)** Expression of AQP2 in HU and REST mice. Kidneys from HU and REST mice were lysed, and immunoblotting experiments were performed using specific antibodies against total AQP2. Densitometric analysis of total AQP2 bands normalized to the total protein content is reported in the histogram. Data are expressed as mean ± S.E.M. (O.D.). Statistical analysis was done using Unpaired t-test (**p < 0.01).

AQP2 expression was therefore evaluated both at mRNA and protein levels, in kidneys from 4 weeks HU mice and compared to Rest mice. Both mRNA (HU: 0.522 ± 0.091 vs. REST: 1.000 ± 0.122; *p = 0.007*) (Figure 4A) and protein levels (HU: 0.625 ± 0.037 O.D. vs. REST: 1.000 ± 0.093 O.D.; *p = 0.002*) (Figure 4B) revealed a significant decrease in AQP2 expression.

In contrast, AQP3 and AQP4 expressions evaluated by Western blotting technique demonstrated no significant alteration after 4 weeks of suspension in HU mice compared to Rest mice (Supplementary Figure S1) indicating a selective downregulation of AQP2.

### HU mice displayed higher levels in AQP2 phosphorylated at Ser261 and AQP2-mRNA targeted miRNA137

Next, the intracellular pathways responsible for AQP2 downregulation were investigated. The regulation of both AQP2 gene and protein expression levels occurs by different regulatory factors: kinases, such as p38MAPK, known as upstream AQP2 degradation, or microRNA, such as miR137, known as a mRNA-AQP2 targeting microRNA. To evaluate the involvement of p38MAPK in regulating the degradation of AQP2, via phosphorylation in Ser261, and of miRNA137 in downregulating the mRNA-AQP2 translation, we performed Western blotting experiments to measure phosphorylated levels of AQP2 (AQP2-pS261) and Phospho-p38MAPK, the active form of this kinase, and RT-PCR assays to compare miR137 levels in HU mice with that in Rest mice.

**FIGURE 5:**
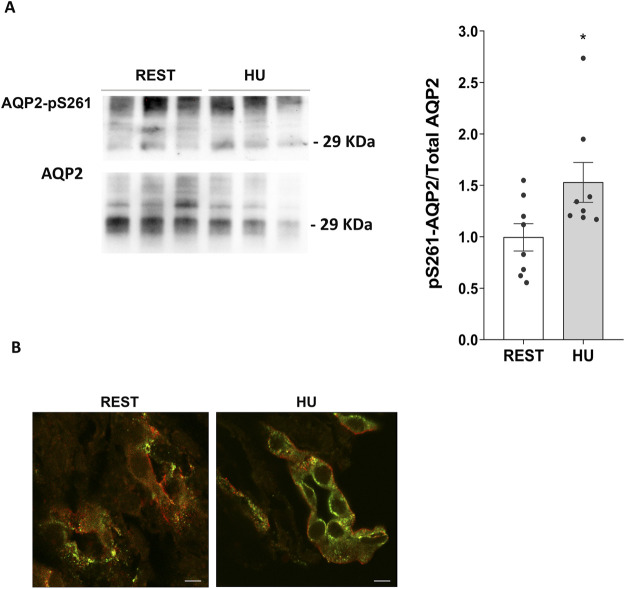
**(A)** Expression of AQP2-pS261 in 4 weeks HU compared to REST mice. Kidneys from HU and REST mice were lysed, and immunoblotting experiments were performed using specific antibodies against phosphorylated AQP2 in Ser261 and total AQP2. Densitometric analysis of AQP2-pS261 bands normalized to total AQP2 is reported in the histogram. Data are expressed as mean ± S.E.M. (O.D.). Statistical analysis was done using Unpaired t-test (*p < 0.05). **(B)** Localization of AQP2-pS261 and total AQP2. Immunofluorescence staining of AQP2-pS261, in green (AlexaFluor 488), and total AQP2, in red (AlexaFluor 555), in renal sections of HU and REST mice (scale bar: 5 μm).

In line to reduced AQP2 expression, 4 weeks HU mice displayed a significant increase in AQP2 phosphorylated at Ser261 (HU: 1.529 ± 0.194 O.D. vs. REST: 1.000 ± 0.133 O.D.; *p = 0.039*) (Figure 5A) suggesting activation of the degradation pathways.

Confocal microscopy and visualization confirmed the decreased expression of AQP2 and the increased phosphorylation levels in Ser261 of AQP2 observed in HU mice kidneys with respect to Rest mice kidneys (Figure 5B).

p38MAPK is a candidate kinase to phosphorylate AQP2 at Serine 261 (Hoffert et al., 2006; Nedvetsky et al., 2010), and its phosphorylation represents a hallmark for ubiquitination and protein degradation via proteasome (Nedvetsky et al., 2010; Isobe et al., 2017).

**FIGURE 6:**
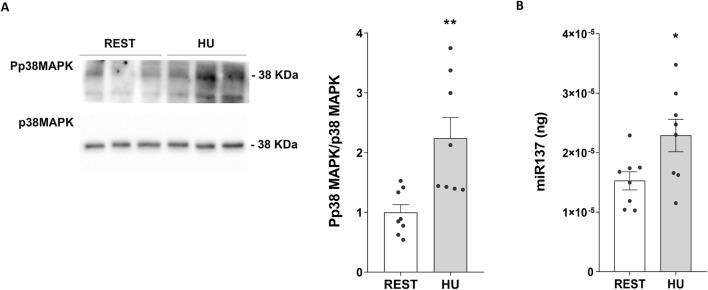
**(A)** Expression of Phosphorylated p38-MAPK (Pp38MAPK) in 4 weeks HU and REST mice. Kidneys from HU and REST mice were lysed, and immunoblotting experiments were performed using specific antibodies against phospho-p38MAPK (Pp38MAPK) and total p38MAPK. Densitometric analysis of Pp38MAPK bands, normalized to total p38MAPK, is reported in the histogram. Data are expressed as mean ± S.E.M. (O.D.). Statistical analysis was done using Unpaired t-test (**p < 0.01). **(B)** Evaluation of miR-137 in HU and REST mice. Total RNA was extracted from HU and REST mice kidneys, and the cDNA was obtained as described in Materials and Methods. Synthetic RNA with 59-phospho, miR-137 (UUAUUGCUUAAGAAUACGCGUAG), was synthesized and used to perform a calibration curve and interpolate miRNA sample values from HU and REST mice. Data from RT-PCR experiments were interpolated in the calibration curve obtained with synthetic miRNA. Data are expressed as mean ± S.E.M. (ng). Statistical analysis was done using Unpaired t-test (*p < 0.05).

Using specific phosphoantibodies recognizing p38MAPK (Pp38MAPK, the active form of the kinase) significantly higher levels of Pp38MAPK were found in HU mice compared to Rest mice (HU: 2.144 ± 0.323 O.D. vs. REST: 1.000 ± 0.118 O.D.; *p = 0.004*) (Figure 6A).

RT-PCR experiments demonstrated higher levels of miRNA137 in HU mice, an AQP2-mRNA targeted miRNA137 (Kim et al., 2015) (HU: 2.290*10^−5^±2.736*10^−6^ ng vs. REST: 1.533*10^−5^±1.522*10^−6^ ng; *p = 0.029*) (Figure 6B).

### AQP2 levels in kidney from HU mice treated with p38MAPK inhibitor SB203580

To understand the actual involvement of p38MAPK in phosphorylating AQP2 and inducing reduced AQP2 expression, we performed experiments exposing kidney slices obtained from HU mice to SB203580 (10 μM for 30 min) a specific inhibitor of p38MAPK.

**FIGURE 7:**
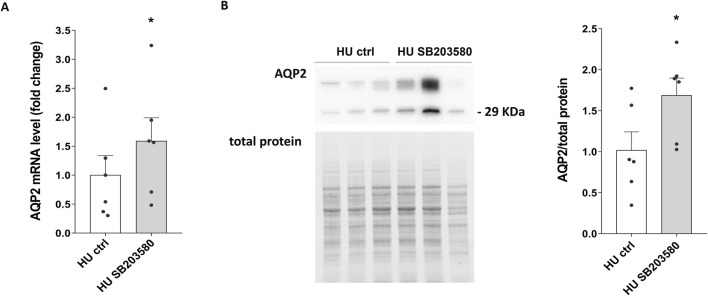
**(A)** AQP2 mRNA levels in 4 weeks HU mice treated with p38MAPK inhibitor SB203580. RNA was extracted from HU mice kidneys left in basal condition or treated with SB203580, as described in Methods Section. Data are expressed as mean ± S.E.M. Statistical analysis was done using paired t-test (*p < 0.05). **(B)** Expression of AQP2 in HU mice treated with the inhibitor SB203580. Kidneys from HU mice were left in basal condition or treated with SB203580 and subsequently lysed. Immunoblotting experiments were performed using specific antibodies against total AQP2. Densitometric analysis of total AQP2 bands normalized to the total protein content is reported in the histogram. Data are expressed as mean ± S.E.M. (O.D.). Statistical analysis was done using paired t-test (*p < 0.05).

Of interest, both AQP2 mRNA (HU SB203580: 1.589 ± 0.402 vs. HU ctrl: 1.000 ± 0.337; *p = 0.013*) (Figure 7A) and protein levels (HU SB203580: 1.686 ± 0.211 O.D. vs. HU ctrl: 1.000 ± 0.224 O.D.; *p = 0.042*) (Figure 7B) were upregulated in kidney slices from HU treated with SB203580 (HU SB203580) compared to kidney slices left under basal condition (HU ctrl), indicating that this kinase is a downstream effector.

**FIGURE 8:**
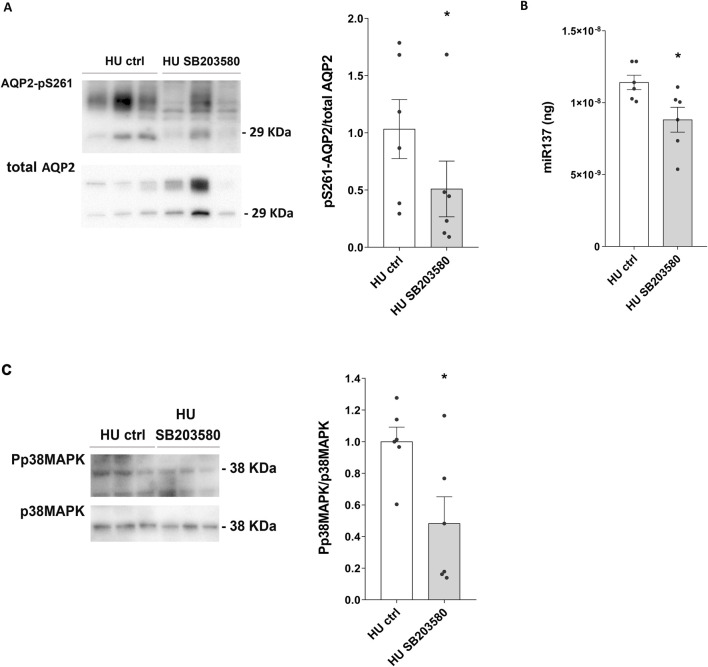
**(A)** Expression of AQP2-pS261 and of Phosphorylated p38-MAPK (Pp38MAPK) in 4 weeks HU mice treated with p38MAPK inhibitor SB203580. HU mice kidneys were left in basal condition or treated with SB203580. Subsequently, kidneys were lysed, and immunoblotting experiments were performed using specific antibodies against AQP2-pS261 and total AQP2. Densitometric analysis of AQP2-pS261 bands normalized to total AQP2 is reported in the histogram. Data are expressed as mean ± S.E.M. (O.D.). Statistical analysis was done using paired t-test (*p < 0.05). **(B)** Evaluation of the effect of SB203580 treatment in HU mice on miR-137 levels. Total RNA was extracted from HU mice kidneys left in basal condition or treated with SB203580, and the cDNA was obtained as described in Materials and Methods. Synthetic RNA with 59-phospho, miR-137 (UUAUUGCUUAAGAAUACGCGUAG), was synthesized and used to perform a calibration curve and interpolate miRNA sample values from HU and REST mice. Data from RT-PCR experiments were interpolated in the calibration curve obtained with synthetic miRNA. Data are expressed as mean ± S.E.M. (ng). Statistical analysis was done using paired t-test (*p < 0.05). **(C)** Expression of Phosphorylated p38-MAPK (Pp38MAPK) in HU mice treated with p38MAPK inhibitor SB203580. Kidneys from HU mice were left in basal condition or treated with SB203580 prior to lysis. Immunoblotting experiments were performed using specific antibodies against phospho-p38MAPK (Pp38MAPK) and total p38MAPK. Densitometric analysis of Pp38MAPK bands, normalized to total p38MAPK, is reported in the histogram. Data are expressed as mean ± S.E.M. (O.D.). Statistical analysis was done using paired t-test (*p < 0.05).

Moreover, a significative reduction in AQP2-pS261 levels was observed in the presence of SB203580 (HU SB203580: 0.510 ± 0.244 O.D. vs. HU ctrl: 1.000 ± 0.259 O.D.; *p = 0.028*) (Figure 8A), suggesting that p38MAPK inhibition prevents AQP2 degradation via phosphorylation at Ser261 in HU mice.

Of note, under these experimental conditions, a significative reduction of the AQP2-mRNA targeted miR137 was observed in HU mice (HU SB203580: 8.815*10^−9^± 8.686*10^−10^ ng vs. HU ctrl: 1.141*10^−8^± 5.008*10^−10^ ng; *p = 0.015*) (Figure 8B).

In line with this, SB203580 reduced phosphorylated p38MAPK levels in HU mice (HU SB203580: 0.482 ± 0.207 O.D. vs. HU ctrl: 1.000 ± 0.113 O.D.; *p = 0.042*) (Figure 8C).

All the described experiments were conducted in mice matched by sex (4 males and 4 females in 4 weeks suspension and 4 males and 3 females in 1 week suspension). No significant difference emerged between the two groups, indicating that the obtained results were apparently not influenced by sex.

## Discussion

The hindlimb unloading (HU) mouse has been confirmed to be a useful model for studying the impact of microgravity or the effects of prolonged immobilization in humans (Globus and Morey-Holton, 2016; Milstead et al., 2004). Exposure to microgravity leads to a redistribution of body fluid to the upper part of the body and an extravasation very early in-flight. These physiological alterations significantly affect renal function, an organ playing a pivotal role in regulating fluid balance, electrolytes, and waste removal in the body. In the absence of gravity, some key changes lead to alterations in how the kidneys function: fluid redistribution, decreased blood volume, altered filtration, electrolyte imbalance and bone loss and calcium metabolism (Olde Engberink et al., 2023). Studying kidney function in microgravity provides valuable insights into how the human body adapts to extreme environments and may offer clues for managing conditions such as kidney disease on Earth or kidney consequences during aging bed rest. In the present contribution vasopressin levels, evaluated for the first time monitoring copeptin, a validated surrogate for plasma vasopressin levels, along with renal aquaporins expression were investigated in HU mice over 4 weeks. Obtained results can be summarized as follows: *a.* vasopressin (copeptin) increased as early as 1 week of unloading which correlated with a significant increase in AQP2 total protein expression and decrease in serum osmolality, suggesting early activation of the vasopressin/AQP2 axis; *b.* at 4 weeks of HU, vasopressin decreased significantly along with reduction of both AQP2 mRNA and AQP2 total protein expression; *c*. the basolateral water channels AQP3 and AQP4 were unaffected.

As mentioned, the observation that vasopressin and AQP2 levels rise at 1 week HU is in agreement with a previous study using HU rat model showing a peak value of both plasma vasopressin levels and AQP2 at HU7 (Chung et al., 2012). Since it is well described that about 10%-15% fluid transfer from the intravascular to interstitial compartment occurs very early in astronauts within the first 24 h (Leach et al., 1996) and in HU rats (Sullivan et al., 2004), this increase in vasopressin might reflect the need to restore vascular fluid loss. In line with that, serum osmolality decreased at 1 week HU. Another study however reported that fluid loss may not occur in spaceflight in quadrupeds such as the rat (Wade and Morey-Holton, 1998). We cannot exclude that stress can also contribute to the observed increase in vasopressin since it is known that stress is often associated with water retention and its resolution with diuresis (Bhattacharya et al., 1998). Nevertheless, the timepoint showing increase in vasopressin is not an early time and it has been reported that HU model is not a stress model (Drummer et al., 2001). We show here that mice suspended for 4 weeks had instead a significant decrease in serum copeptin, associated with reduced expression of AQP2 both in terms of mRNA and protein levels. Functional alterations of vasopressin observed in the HU model are compatible with an adaptation to the fluid redistribution towards the cephalic part of the body, causing central hypervolemia and consequently a reduction of vasopressin. We observed that the reduction in vasopressin levels was paralleled by the downregulation of AQP2 expression. These effects are expected to limit fluids reabsorption, counteracting central hypervolemia. To our knowledge this is the first study evaluating vasopressin levels in HU mice suspended for 4 weeks. As discussed, a previous study using HU rat model showed a peak value of plasma vasopressin (measured with RIA method) after 1 week associated with reduction in AQP2 expression. These alterations were however restored after 2 weeks (Chung et al., 2012), and the authors suggest that, in this model, vasopressin system may require 2 weeks for adaptation to microgravity. In contrast, in HU mice evaluated in our study, vasopressin levels remained significantly lower with respect to the starting timepoint. If 4 weeks suspension can be considered a period of chronic adaptation microgravity, then our results differ from data in astronauts during long-term spaceflights displaying instead increased levels of vasopressin (Drummer et al., 2000; Grigoriev et al., 1994) despite no change in plasma osmolality that can be the consequence of decreased plasma volume. We cannot explain this discrepancy that can be related to a different behavior of mice with respect to humans. In addition, it is possible that body fluid regulation in microgravity in space differs from that on earth as suggested by other studies (Drummer et al., 2000). In fact, in humans, renal responses during exposure to simulated microgravity are consistently weaker than those during simulation experiments before spaceflight (Norsk et al., 2000).

HU causes a cephalad fluid shift and removal of ground reaction forces from the hindlimbs as occurs in astronauts. However, gravity is still present in the HU model with a headward shift of internal organs and fluid. The internal organs still exert pressure against each other with HU, unlike in spaceflight, during which organs free float.

In our study we provide evidence that the downregulation of the vasopressin-sensitive water channel AQP2 is mainly the consequence of activation of its degradation. AQP2 trafficking and expression are fine controlled by various post-translational events, like phosphorylation, ubiquitination, and degradation (Moeller et al., 2011; Ranieri et al., 2018), and glutathionylation (Tamma et al., 2014). The binding of vasopressin to its receptor causes AQP2 phosphorylation at Serine 256, 264 and 269 at short term; while, at long term, AQP2 mRNA expression increases and, consequently, rises AQP2 protein level (Matsumura et al., 1997; Fenton et al., 2008; Hoffert et al., 2008). On the other hand, under vasopressin stimulation, phosphorylation at Serine 261 of AQP2 decreases (Hoffert et al., 2006; Matsumura et al., 1997; Hoffert et al., 2007; Nedvetsky et al., 2010). Both events, AQP2 phosphorylation at Serine 261 and polyubiquitination induce AQP2 internalization, recycling or degradation via p38MAPK (Nedvetsky et al., 2010; Trepiccione et al., 2014). p38MAPK is a candidate kinase to phosphorylate AQP2 at Serine 261 (Hoffert et al., 2006; Nedvetsky et al., 2010), and its phosphorylation represents a hallmark for ubiquitination and protein degradation via proteasome (Nedvetsky et al., 2010; Isobe et al., 2017).

HU mice showed a significant increase in the Serine 261 phosphorylation of AQP2. This increase in phosphorylation is crucial for the degradation processes of AQP2 causing the decrease of the AQP2 protein levels. We provide here evidence that phosphorylation at Serine 261 is mediated by p38MAPK activation.

We previously demonstrated that p38MAPK could activate miRNAs transcription via ATF1 transcription factor (Ranieri et al., 2024). miR137 is a microRNA, a small non-coding RNA molecule, involved in the regulation of AQP2 gene expression. MicroRNAs typically function by binding to complementary sequences on target messenger RNAs (mRNAs), usually resulting in their silencing through translational repression or target degradation.

Recent studies have started to uncover the relationship between miR137 and AQP2, suggesting that miR137 may influence AQP2 expression and thereby affect kidney function and water balance (Kim et al., 2015; Ranieri et al., 2018; Ranieri, 2019; Ranieri et al., 2024). miR137 may directly target the mRNA of AQP2, leading to its degradation or inhibition of translation. Interestingly we show here that in HU mice miR137 increases resulting in decreased levels of AQP2 protein. Of note, p38MAPK inhibition with SB203580 restores miR137 levels in HU mice compared to Rest mice, demonstrating that this kinase is responsible for miR137 transcription.

This result shows how, by regulating AQP2 levels, miR137 can influence water reabsorption in the collecting ducts of the kidney. Lower levels of AQP2 due to high miR137 activity could lead to reduced water reabsorption and increased urine output. However, we could not measure urinary output in HU mice in this study.

At renal level we have already demonstrated that miR137 levels are increased in parallel with CaSR activation. CaSR stimulation promotes p38MAPK activation able to activate the transcription factor ATF1, which in turn causes an increase in miR137 transcription (Ranieri et al., 2024).

The activation of CaSR is possibly due to an increase in calciuria secondary to an increase in serum calcium concentration, due to bone reabsorption (Rittweger et al., 2009; Bilancio et al., 2013). Unfortunately, because urines were not collected from HU mice, calciuria could not be measured. However, we already demonstrated in these same HU mice that bone from control and HU mice at 4 week had decreased thickness of trabeculae and increasing number of empty lacunae reflecting bone demineralization (Sanesi et al., 2023) which is expected to result in increased calciuria. Increased urinary calcium can activate CaSR which in turn counteracts vasopressin-AQP2 response resulting in reduction of urinary AQP2 excretion (Procino et al., 2012; Valenti et al., 2000) as also observed in HU mice at 4 weeks.

**FIGURE 9:**
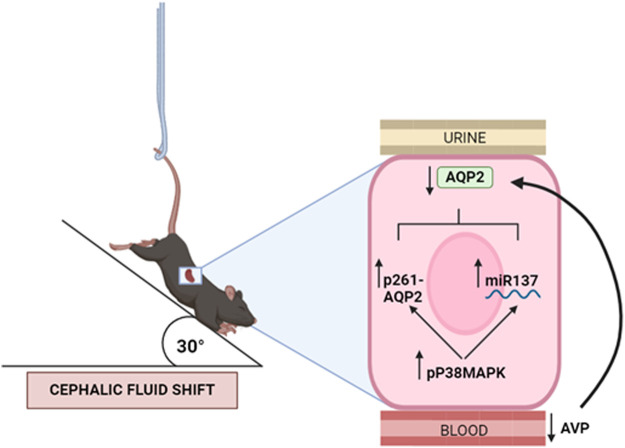
Proposed model of kidney mice adaptation to 4 weeks hindlimb suspension (HU). HU suspension is associated with reduced secretion of vasopressin (copeptin) hormone and selective reduction of AQP2 expression, with no change in the expression of other renal AQPs. The AQP2 downregulation occurs through an increased activity of p38MAPK which in turn promotes internalization and degradation via phosphorylation of AQP2 in Ser261 and decreases the AQP2 mRNA translation via miR137.

In conclusion, as summarized in the schematic model in Figure 9, this study demonstrates that chronic adaptation to microgravity of mice, simulated by 4 weeks HU suspension, induces a reduced secretion of vasopressin and selective reduction of AQP2 expression mainly due to its degradation, with no change in the expression of other renal AQPs. This response is likely the consequence of the cephalic shift of fluids caused by HU suspension and represents a physiological adaptation to counteract the central hypervolemia.

## Materials and methods

### Antibodies

AQP2 ((E-2) sc-515770), AQP3 ((C-18) sc-9885) and AQP4 ((C-19) sc-9888) antibodies were purchased from Santa Cruz Biotechnology (Tebu Bio, Milan, Italy). AQP2-pS261 (AQP2 (pSer261) NB100-61100) antibody was from Novus Biologicals (Littleton, CO, United States).

p38MAPK (9212) and phospho-p38 MAPK (Thr180/Tyr182; #9211S) antibodies were obtained from Cell Signaling Technologies Inc., Beverly, MA.

Secondary goat anti-rabbit and mouse antibodies conjugated to horseradish peroxidase (HRP) were obtained from Merck (Merck KGaA, Darmstadt, Germany), whereas secondary donkey anti-goat antibody conjugated to horseradish peroxidase (HRP) was purchased from Santa Cruz Biotechnology (Tebu Bio, Milan, Italy). Secondary goat anti-rabbit Alexa 488 and anti-mouse Alexa 555 conjugate antibodies were from Molecular Probes (Eugene, OR, United States).

### Animal models

Hind-limb suspended C57BL6 male and female mice were subjected to the tail suspension technique, according to recommendations by Morey-Holton et al. (2005) and as previously described (Sanesi et al., 2023). Mice were suspended to prevent any contact of the hind limbs with the cage floor, maintaining approximately a 30° head-down tilt. The forelimbs of animals were in contact with the cage bottom to guarantee full access to the entire cage. Each mouse was singly housed, with access to water and regular diet ad libitum (Harlan Teklad, 2019, SDS, England) and maintained under standard conditions on a 12/12 h light/dark cycle. 8-Week-old male and female mice C57BL6 were randomly assigned to two groups: mice kept in control condition (4 weeks Rest, n = 8, four males and four females) and hind-limb unloading suspended mice (4 weeks HU, n = 8, four males and four females) and mice kept in control condition (1 week Rest, n = 7, four males and three females) and hind-limb unloading suspended mice (1 week HU, n = 7, four males and three females). Mice were weighed once a week and at the end of the experimental procedures were euthanized. Both left and right kidney were surgically excised and stored at −80°C until analysis.

This animal interventional study is in accordance with the European Law Implementation of Directive 2010/63/EU and all experimental protocols were reviewed and approved by the Veterinary Department of the Italian Ministry of Health (Project 522- 2016PR).

### Serum copeptin measurement

Serum copeptin levels were quantified in this study. To obtain the serum samples, blood was collected, and the tubes were immediately placed on ice and subjected to centrifugation at 1,600 × g for 15 min at 4°C. This centrifugation process facilitated the separation of serum from other blood components. The collected serum samples were promptly stored at a temperature of −80°C until they were ready for further analysis.

The levels of Copeptin (CPP) were measured using an ELISA kit (Cloud-Clone Corp., TX, United States). This kit offers high sensitivity and excellent specificity for copeptin detection, with a detection limit lower than 9.6 pg/mL.

### Serum Osmolality measurement

Serum Osmolality was measured by using VAPRO® Vapor Pressure Osmometer (Wescor Model 5,520; Wescor Inc., United States).

### Tissue preparation

Studies in kidney slices were performed as described (Boone and Deen, 2008). In brief, kidneys were quickly excised and thin traversal slices (500 μm), including cortex and medulla, were obtained using a stereomicroscope. Kidney sections were then equilibrated in a kidney slices buffer (118 mM NaCl, 16 mM HEPES, 17 mM Na-HEPES, 14 mM Glucose, 3.2 mM KCl, 2.5 mM CaCl_2_, 1.8 mM MgSO_4_, and 1.8 mM KH_2_PO_4_, pH 7.4) for 10 min. The kidney slices were maintained at 37°C untreated or treated with 10 μM SB203580 for 30 min. Subsequently, the kidney slices were homogenized in ice-cold kidney slices buffer supplemented with protease (1 mM PMSF, 20 μM leupeptin and 2 μg/mL pepstatin A) and phosphatase (10 mM NaF and 1 mM sodium orthovanadate) inhibitors, using a mini potter. Suspensions were then centrifuged at 12,000 x g for 10 min at 4°C and the supernatants used for Western blotting analysis.

### Gel electrophoresis and immunoblotting

60 μg of proteins were separated using 12% stain-free polyacrylamide gels (Bio-Rad Laboratories, Inc., Hercules, CA, United States) under reducing conditions. Protein bands were electrophoretically transferred onto Immobilon-P membranes (Millipore Corporate Headquarters, Billerica, United States) for Western blot analysis. These membranes were blocked in TBS-Tween-20 solution containing 3% bovine serum albumin (BSA) and incubated overnight with primary antibodies. anti-AQP2, anti-pS261AQP2, anti-AQP3, anti-AQP4, anti-p38MAPK, and anti-Pp38MAPK were used at 1:500 dilution.

Immunoreactive bands were detected using secondary antibodies (goat anti-rabbit, goat anti-mouse and donkey anti-goat) conjugated to horseradish peroxidase (HRP), which were obtained from Santa Cruz Biotechnologies (Tebu-Bio, Milan, Italy). Membranes were developed using Clarity Western ECL Substrate with the ChemiDoc System gels (Bio-Rad Laboratories, Milan, Italy).

Obtained bands were normalized to total protein using stain-free technology gels. Densitometry was performed using Image Lab software (Bio-Rad Laboratories, Milan, Italy), and the data were analyzed using GraphPad Prism (GraphPad Software, San Diego, CA, United States).

### Isolation of total RNA, reverse transcription, and real-time PCR analysis of mRNA of AQP2 in REST and HU mice

To evaluate the expression level of mRNA of AQP2 in the kidney papillae isolated from REST and HU mouse, real-time PCR experiments were conducted. Total RNA was extracted from the samples using Trizol method (Thermo Fisher Scientific). Reverse transcription was performed on 2 μg of total RNA using SuperScript Vilo Master Mix (Invitrogen, Life Technologies, Monza, Italy). Real-time PCR amplification was carried out using TaqMan Gene Expression PCR Master Mix with AQP2 assay (Assay ID: Mm00437575_m1), using 18S assays (Assay ID: Hs99999901_s1) as housekeeping gene in the StepOne Real-Time PCR System (Thermo Fisher Scientific, Waltham, MA, United States), setting the thermal cycling conditions as specified by the manufacturer (95°C for 20 s; 40 cycles alternatively at 95°C for 1 s and 60°C for 20 s). Results were quantified as 2^−ΔΔCt^ values, representing the relative expression levels. The calculation of ΔΔCt was based on the following formula: ΔΔCt = (Ct_target_–Ct_18S_)_HU_ - (Ct_target_–Ct_18S_)_REST_.

### Isolation of total RNA, reverse transcription, and real-time PCR analysis of miRNA-137 in REST and HU mice

The miRNA-137 levels in the kidney papillae of REST and HU mice were assessed using TaqMan Advanced miRNA Assays (has-miR-137; Assay ID: 477904_mir; Applied Biosystems), which provided highly sensitive and specific quantification of mature miRNA through quantitative PCR. Total RNA was extracted from the samples using Trizol method (Thermo Fisher Scientific). Reverse transcription was performed on 5 ng of total RNA using the TaqMan Advanced miRNA cDNA Synthesis Kit (Applied Biosystems). A calibration line was generated using a synthetic RNA (UUAUUGCUUAAGAAUACGCGUAG) with a 5′-phosphate, synthesized by Applied Biosystems, to accurately evaluate the miR137 content (in ng) in the samples from REST and HU mice by interpolating the miRNA values.

#### Immunofluorescence

Mouse kidneys were fixed overnight by immersion in 4% (w/v) paraformaldehyde at 4°C, washed in PBS and cryopreserved in 30% (w/v) sucrose in PBS at 4°C for 24 h, and then embedded in paraffin medium. Thin sections (5 μm) were subject to immunofluorescence analysis as follows: after blocking with 1% BSA in PBS for 20 min at room temperature, sections were incubated overnight at 4°C with primary antibodies anti-AQP2 and anti-pS261AQP2. The following day sections were washed three times with PBS and stained with 1:1,000 donkey anti-rabbit Alexa Fluor 488 (Thermo Fisher Scientific, Waltham, MA, United States) or with 1:1,000 anti-mouse Alexa Fluor 555 (Thermo Fisher Scientific, Waltham, MA, United States) for 1 h at room temperature. Next, slides were washed three times with PBS and coverslips were applied onto them, using Mowiol Mounting Medium (Merck KGaA, Darmstadt, Germany). Confocal images were obtained with a confocal laser-scanning fluorescence microscope (Nikon AX R).

### Statistical analysis

Statistical analysis was performed using unpaired parametric two-tailed t-test and paired two-tailed t-test for ctrl vs. SB203580 experiments. All values are presented as a means with their corresponding standard error medium (S.E.M.). The difference of *p < 0.05* was considered statistically significant.

## Data Availability

The raw data supporting the conclusions of this article will be made available by the authors, without undue reservation.

## Supplementary material

SUPPLEMENTARY FIGURE 1 Expression of AQP3 and AQP4 in 4 weeks HU and REST mice. Kidneys from HU and REST mice were lysed, and immunoblotting experiments were performed using specific antibodies against AQP3 and AQP4. Densitometric analysis of AQP3 and AQP4 bands normalized to the total protein content is reported in the histogram. Data are expressed as mean ± S.E.M. (O.D.). Statistical analysis was done using Unpaired t-test.
