# Supplementary material for: Alteration of vasopressin-aquaporin system in hindlimb unloading mice
Source: Front Physiol. 2025 Apr 15;16:1535053. doi: 10.3389/fphys.2025.1535053 (PMC12037502; doi:10.3389/fphys.2025.1535053)
Supplement: Supplementary file 1 [file DataSheet1.zip › Supplementary original WBs.pptx]

## Slide 1
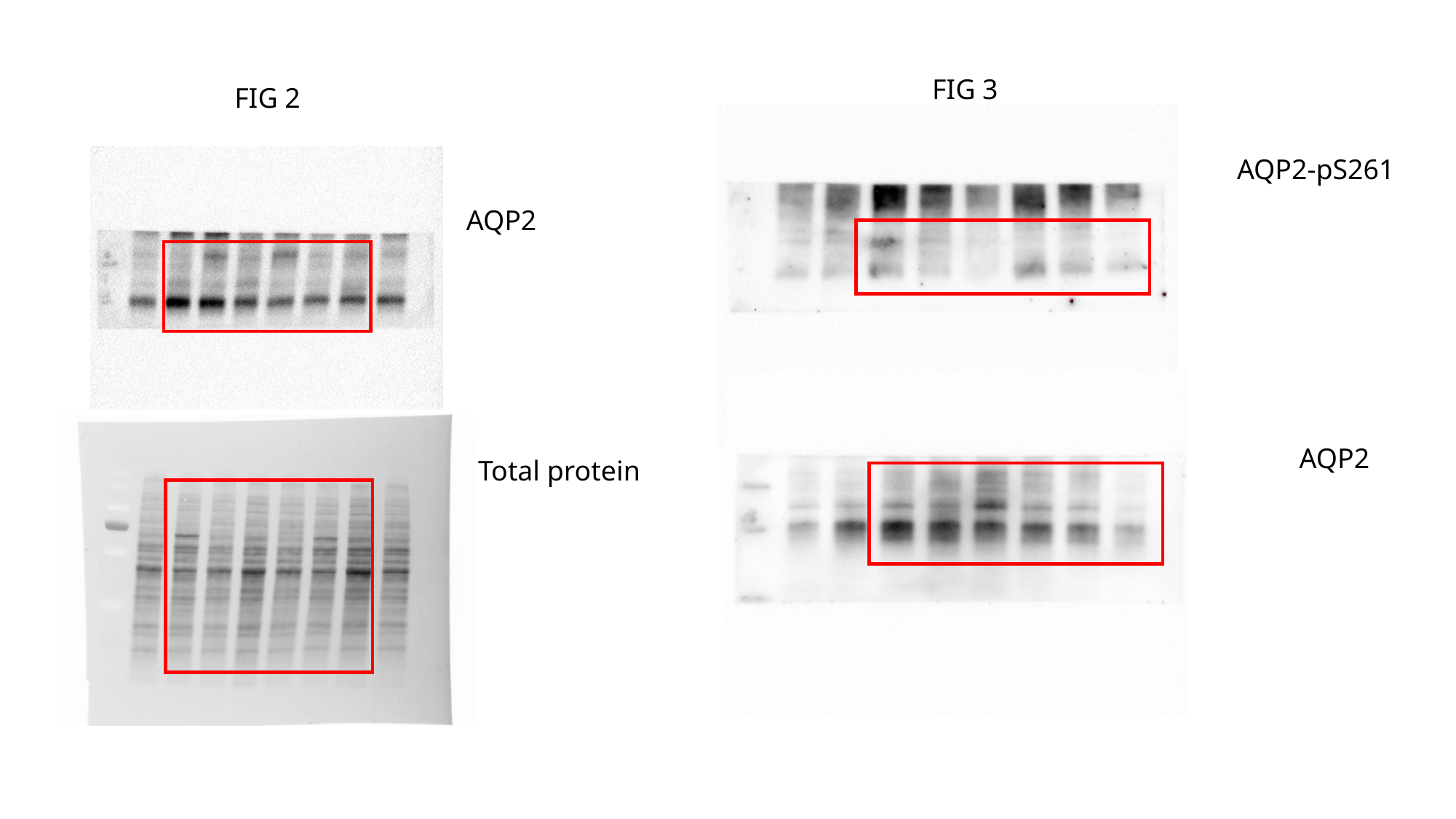

FIG 3
FIG 2
AQP2-pS261
AQP2
AQP2
Total protein

## Slide 2
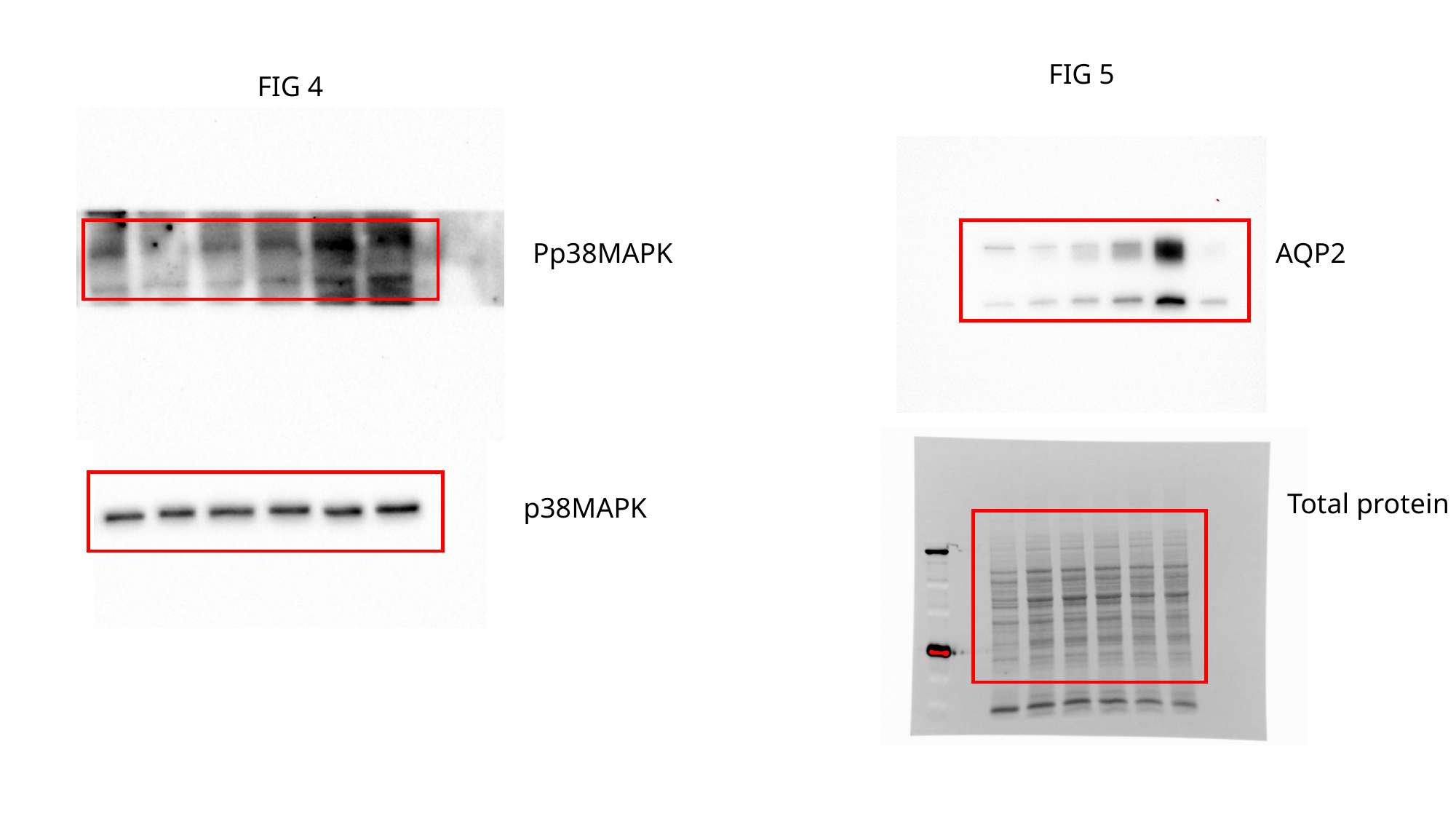

FIG 5
FIG 4
Pp38MAPK
AQP2
Total protein
p38MAPK

## Slide 3
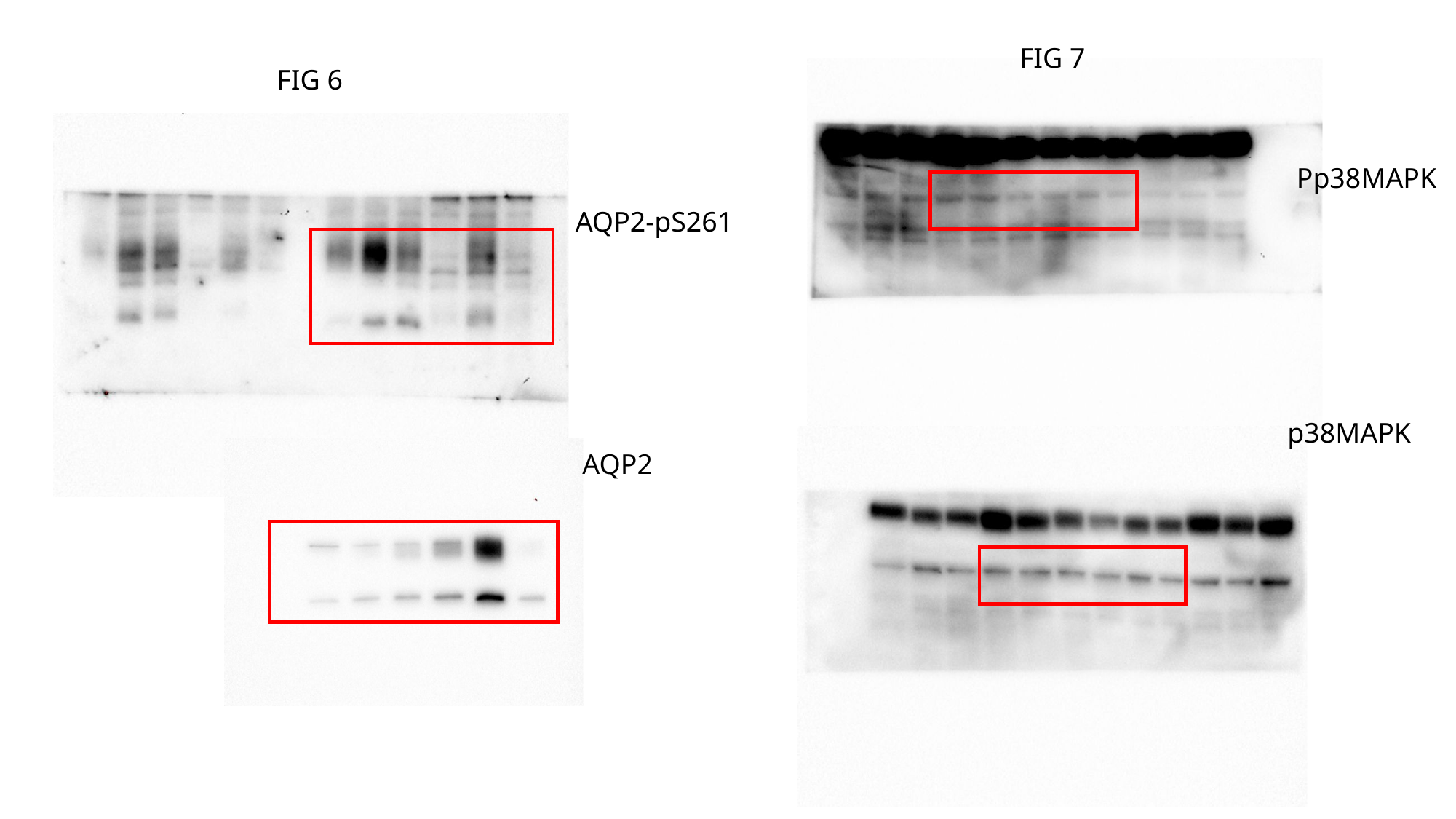

FIG 7
FIG 6
Pp38MAPK
AQP2-pS261
p38MAPK
AQP2
