# Supplementary figures and images for: Alteration of vasopressin-aquaporin system in hindlimb unloading mice
Source: Front Physiol. 2025 Apr 15;16:1535053. doi: 10.3389/fphys.2025.1535053 (PMC12037502; doi:10.3389/fphys.2025.1535053)

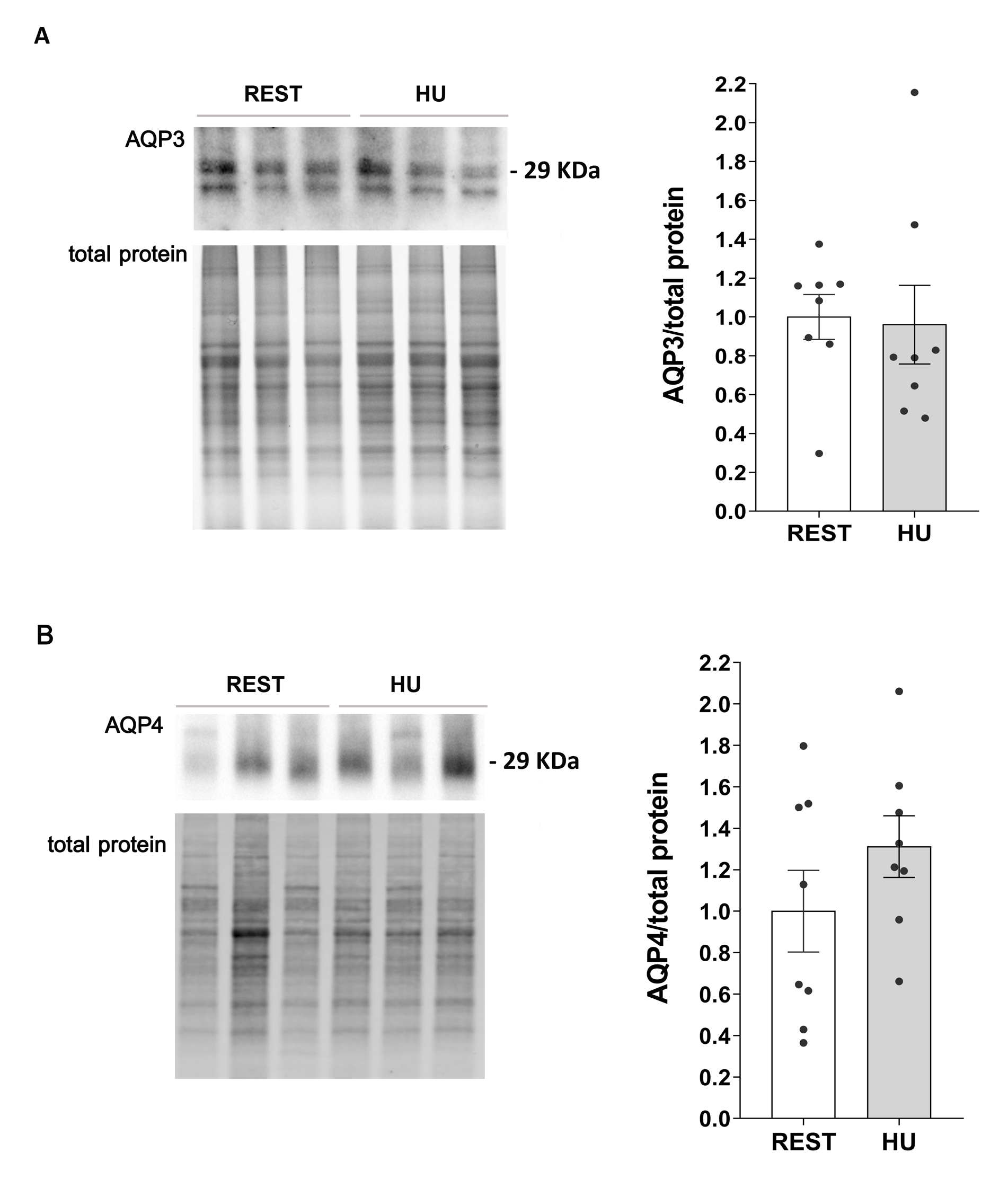

Supplement: Supplementary file 2 [file Image1.tif]
